# Identification of cellular ion channels that facilitate Hazara nairovirus infection enables selection of clinically approved compounds with anti-nairoviral properties

**DOI:** 10.1038/s41598-026-42810-7

**Published:** 2026-03-24

**Authors:** Frank W. Charlton, Samantha E. Hover, Aseel Alyahyawi, Hayley M. Pearson, Thomas A. Edwards, Jamel Mankouri, Martin Stacey, Juan Fontana, John N. Barr

**Affiliations:** 1 School of Molecular and Cellular Biology, Faculty of Biological Sciences, University of Leeds, Leeds, LS2 9JT UK; 2 Astbury Centre for Structural and Molecular Biology, University of Leeds, Leeds, LS2 9JT UK; 3 Present Address: Laboratory of Environmental Virology (LEV), l’École Polytechnique Fédérale de Lausanne, Lausanne, Switzerland; 4 Present Address: College of Biomedical Sciences, Larkin University, 18301 N Miami Avenue, Miami, FL 33169 USA; 5 Instituto Biofisika (IBF), CSIC-UPV/EHU, Barrio Sarriena s/n, 48940 Leioa, Spain

**Keywords:** Drug discovery, Microbiology

## Abstract

The *Nairoviridae* family of segmented negative-sense RNA viruses includes the serious human pathogen Crimean-Congo haemorrhagic fever virus (CCHFV), associated with a case/fatality rate of up to 40% for which no approved vaccines or treatments exist. Nairoviruses internalize via endocytosis and pass through the endolysosomal network, exploiting the changing ionic environment to promote envelope fusion. Fusion is influenced by hydrogen (H^+^) and potassium ions (K^+^), which increase in concentration as endosomes mature, regulated by host ion channels. Using the model nairovirus Hazara virus (HAZV) of the CCHFV serogroup, we performed an siRNA screen to identify cellular ion channels involved in nairovirus infection. Most high-ranking hits belonged to K^+^ and calcium (Ca^2+^) channel families. Consistent with this, we showed that clinically-approved K^+^ channel blockers quinidine, quinine and dronedarone and clinically-approved Ca^2+^ channel blockers tetrandrine and nifedipine significantly reduced HAZV activities. To further probe the role of K^+^ in HAZV infection, we used time-of-addition studies, showing K^+^ was required during entry. Biochemical experiments showed K^+^ expanded the pH range that promoted entry, potentially allowing endosome escape deeper within the endolysosomal network. These results show clinically-approved channel blockers effectively inhibit HAZV replication, suggesting repurposing existing therapies may represent promising avenues to block nairovirus infection.

**Supplementary Information:**

The online version contains supplementary material available at 10.1038/s41598-026-42810-7.

## Introduction

Hazara virus (HAZV) is a tick-borne member of the *Nairoviridae* family, *Hareavirales* order, *Bunyaviricetes* class. HAZV virions are enveloped and contain a tri-segmented, negative-sense, single-stranded RNA genome. These three RNA segments, termed small (S), medium (M) and large (L), encode for the viral nucleoprotein (N), glycoproteins (Gn and Gc) and polymerase (L), respectively. HAZV belongs to the same serogroup as the highly pathogenic Crimean-Congo haemorrhagic fever virus (CCHFV)^1^, which can cause lethal haemorrhagic fever in humans, with fatality rates of up to 40%, and for which there are no preventive or therapeutic measures^2^. Therefore, HAZV represents a tractable surrogate system that can be studied at bio-safety level (BSL) 2, in contrast to CCHFV, which requires BSL4 containment for study. As such, an improved understanding of how HAZV infection can be blocked has the potential to inform CCHFV disease prevention strategies.

While not approved by regulatory authorities, different treatments for CCHFV-mediated disease have been examined^3^. These include the use of nucleoside analogs to disrupt viral RNA synthesis, although such efforts have either shown poor efficacy in the clinic (for example, ribavirin^4^) or require further clinical trials (for example, favipiravir^5,6^). Additional proposed interventions include the use of monoclonal antibodies^7^ or convalescent plasma from survivors^8^, but these approaches also require further pre-clinical and clinical studies. Interestingly, neutralizing antibodies targeting the CCHFV envelope spike proteins do not necessarily confer complete protection in challenge studies, with antibodies directed towards the internal N protein shown to elicit potent protection^9^. Remarkably, N antibodies elicit their protection intracellularly, based on recognition by the TRIM21 Fc receptor^10^, thus opening the possibility of directing therapeutic antibodies against internal viral components. Attempts have also been made involving the use of corticosteroids to alleviate infection, although limited data is available to evaluate their efficacy^11^.

A promising target for antiviral therapies is the process of virus entry. As with many enveloped viruses, entry of nairoviruses into the host cell involves internalization within compartments of the endolysosomal system, where conformational changes in the nairoviral glycoproteins^12^ are triggered by the changes in lumenal H^+^ and K^+^ concentrations as endosomes mature. Such spike structural changes are required for the fusion of the viral and cellular membranes and the release of the viral genome into the cytoplasm for subsequent gene expression, thus demonstrating a viral dependence on host factors for entry^13^. The ionic changes within the endolysosomal network are largely controlled by ion channels, and it is important to note that approximately 19% of current FDA-approved drugs are ion channel modulators^13^. It follows that modulation of such channels offers the potential to alter the ionic composition within endolysosomal compartments^14^, thus potentially influencing the process of virus entry. Repurposing clinically approved drugs represents a promising avenue to treat viral infections, given these drugs have already been shown to be safe for humans.

Here, to identify cellular ion channels involved in nairovirus multiplication, we subjected HAZV to a screen using a curated library of 264 siRNAs directed against 88 cellular ion channels, which revealed that the majority of high-ranking hits were members of the K^+^ and Ca^2+^ ion channel families. Consistent with this finding, both broad- and narrow-spectrum clinically-approved K^+^ channel blocking drugs, including dronedarone (Dron), quinidine (Qn) and quinine (Qn), and Ca^2+^ channel drugs tetrandrine (Tet) and nifedipine (Nif) significantly reduced HAZV growth in human-derived cells. To identify the stage within the replication cycle at which K^+^ channel blockade influenced HAZV activities, we performed time-of-addition studies, showing K^+^ was required during HAZV entry. To probe the role of K^+^ in the entry process, we performed biochemical in vitro experiments, revealing increased K^+^ concentrations expanded the pH range that promoted efficient infection from pH 7.3 to pH 6.9, potentially allowing fusion deeper within the endosomal network. Taken together, these results suggest that repurposing existing approved therapies may represent promising avenues to block nairovirus infection.

## Results

### Multiple ion channels support HAZV multiplication

**Fig. 1:**
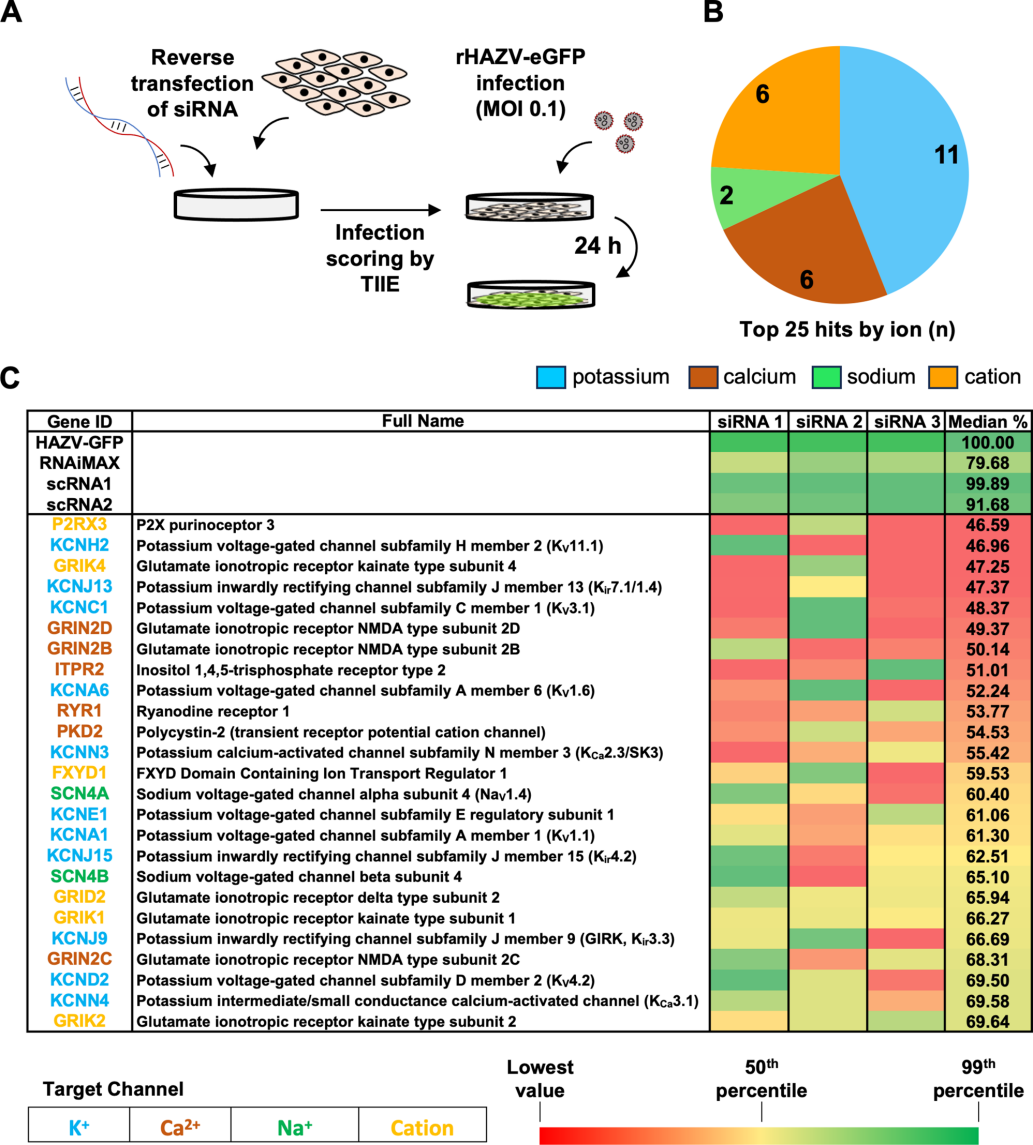
Cellular ion channels are required for HAZV infection. (**A**) Workflow of siRNA screening. A549 cells were reverse-transfected with one of three siRNAs targeting the indicated ion channels and incubated at 37°C for 48 h. Cells were then infected with rHAZV-eGFP (MOI 0.1) for 24 h, when whole-well images of cells were taken using the IncuCyte S3 system using the phase contrast and green fluorescence optics. Infection was scored using TIIE and normalised to cell confluency to account for potential cytotoxicity. Treatment with scrambled RNAs (scRNA) and incubation with transfection reagent only (RNAiMAX) were used as controls. (**B**) Number of most abundant ion channel types in the top 25 hits. (**C**) Summary data of siRNA screening results, showing % of TIIE (infection) compared to untreated cells. Transfection reagent (RNAiMAX) only and scrambled RNA (scRNA) 1 and 2 controls are shown. Colour scale corresponds to % knockdown, with values in the 99th percentile shown green, 50th percentile shown in yellow and the lowest percentile shown in red. n = 2, as two technical replicates of two biological replicates.

Building on previous reports suggesting HAZV requires elevated endosomal K^+^ concentrations for entry^12^, we employed rHAZV-eGFP^15^ in a high-throughput screen to identify host cell ion channels that support HAZV infection. Specifically, we utilised a library of 264 siRNAs targeting 88 human ion channels, including K^+^, Na^+^ and Ca^2+^ channels, with three unique siRNAs targeting each channel (Fig. 1A)^16^. A549 cells were reverse-transfected with siRNAs and infected with rHAZV-eGFP (MOI 0.1), with virus-specific gene expression assessed using live-cell analysis by measuring total integrated intensity of eGFP (TIIE) at 24 h post infection (hpi), taking advantage of the free eGFP expressed by this virus (Fig. 1A). This 24-h assay period, represents more than one infectious cycle, thus measurement of gene expression in this way serves to assess all stages of the infection cycle including entry, transcription, genome replication, translation, assembly, egress and also encompassing gene expression in newly infected cells. The effect of each siRNA was assessed 4 times, by way of two technical repeats of two biological repeats, and the median TIIE for each of the three siRNAs was normalised to that of non-transfected rHAZV-eGFP infected cells. Summary results for the top 25 gene identities associated with the largest reduction in TIIE are presented both as a pie-chart showing channel family abundance (Fig. 1B), and as a table displaying the median TIIE reduction for each siRNA (Fig. 1C). Individual TIIE data points are also shown plotted as histograms (Supplementary Fig. S1) and presented in a spreadsheet (Supplementary Data Set 1).

Of the top 25 gene targets whose knockdown inhibited HAZV infection, 11 were K^+^ channels, 6 were Ca^2+^ channels, 2 were Na^+^ channels and the remaining 6 were non-selective cation channels (Fig. 1B, C and Supplementary Fig. S1). Of the most abundant K^+^ channel group, knockdown of the KCNH2 and KCNJ13 genes, encoding the voltage-gated K^+^ channel K_v_11.1 and the inwardly-rectifying K^+^ channel K_ir_7.1 respectively, reduced median TIIE to approximately 47% that of untreated rHAZV-eGFP-infected cultures. Similarly, knockdown of K_V_ encoding genes KCNC1 (K_v_3.1) and KCNA6 (K_v_1.6) also appeared in the top 10 hits. Several Ca^2+^ channels were also highly ranked in our screen, with 4 representatives within the top 10 hits, with TIIE reduced to around 50% of untreated cells. In contrast, the two Na^+^ channels within the top 25 showed a more modest reduction in TIIE, to around 60–65%. Taken together, these results suggest that the activity of multiple ion channels influence the HAZV infection process.

### HAZV is sensitive to blockade of Ca^2+^ channels, but not Na^+^ channels

Ca^2+^ channels have previously been shown to support bunyavirus multiplication, with Hantaan hantavirus^17^ and severe fever with thrombocytopenia syndrome virus (now Dabie bandavirus) reliant on Ca^2+^ channel function for productive infection^18^. Interestingly, the results of the siRNA screen (Fig. 1B and C) suggested 6 Ca^2+^ channels played an important role in HAZV gene expression. To confirm a role for these channels, we next examined the effect of broad-spectrum Ca^2+^ channel blockers on HAZV activities. First, A549 cells were pretreated with Tet, a known inhibitor of voltage-dependent Ca^2+^ channels with proposed antiviral potential^19,20^ and infected with rHAZV-WT, with subsequent HAZV gene expression assessed at 24 hpi by western blotting for HAZV N protein (Supplementary Fig. 2A). Quantification of blots showed a step-wise decrease in response to increased inhibitor concentrations, with the highest concentration reducing N protein abundance to around 60% that of untreated cells (Supplementary Fig. 2B). These findings were confirmed by infecting Tet pre-treated cells with rHAZV-eGFP, with eGFP fluorescence measured by live cell imaging (Supplementary Fig. 2C and D). Broadly similar results were obtained using the Ca^2+^ channel blocker Nif, a dihydropyridine clinically-approved as an antihypertensive and anti-anginal medication^21,22^, with the highest concentration reducing N protein abundance to around 40% that of untreated cultures (Supplementary Fig. 2E and F), with a similar drop in eGFP expression from rHAZV-eGFP (Supplementary Fig. 2G and H). For all inhibition assays, non-toxic concentrations were determined by MTS assay (Supplementary Fig. 2I and J).

The siRNA screen also suggested two Na^+^ channels influenced HAZV processes (Fig. 1C). To corroborate this finding, we tested whether the highest non-toxic concentrations of Na^+^ channel blockers lidocaine, procainamide and disopyramide, all class I sodium channel blockers^23^, affected HAZV gene expression (Supplementary Fig. 3). rHAZV-eGFP was used to infect A549 cells pretreated with each inhibitor, with TIIE used to score infection. However, no significant change in TIIE was detected.

### HAZV infection is sensitive to K^+^ channel blockade

**Fig. 2:**
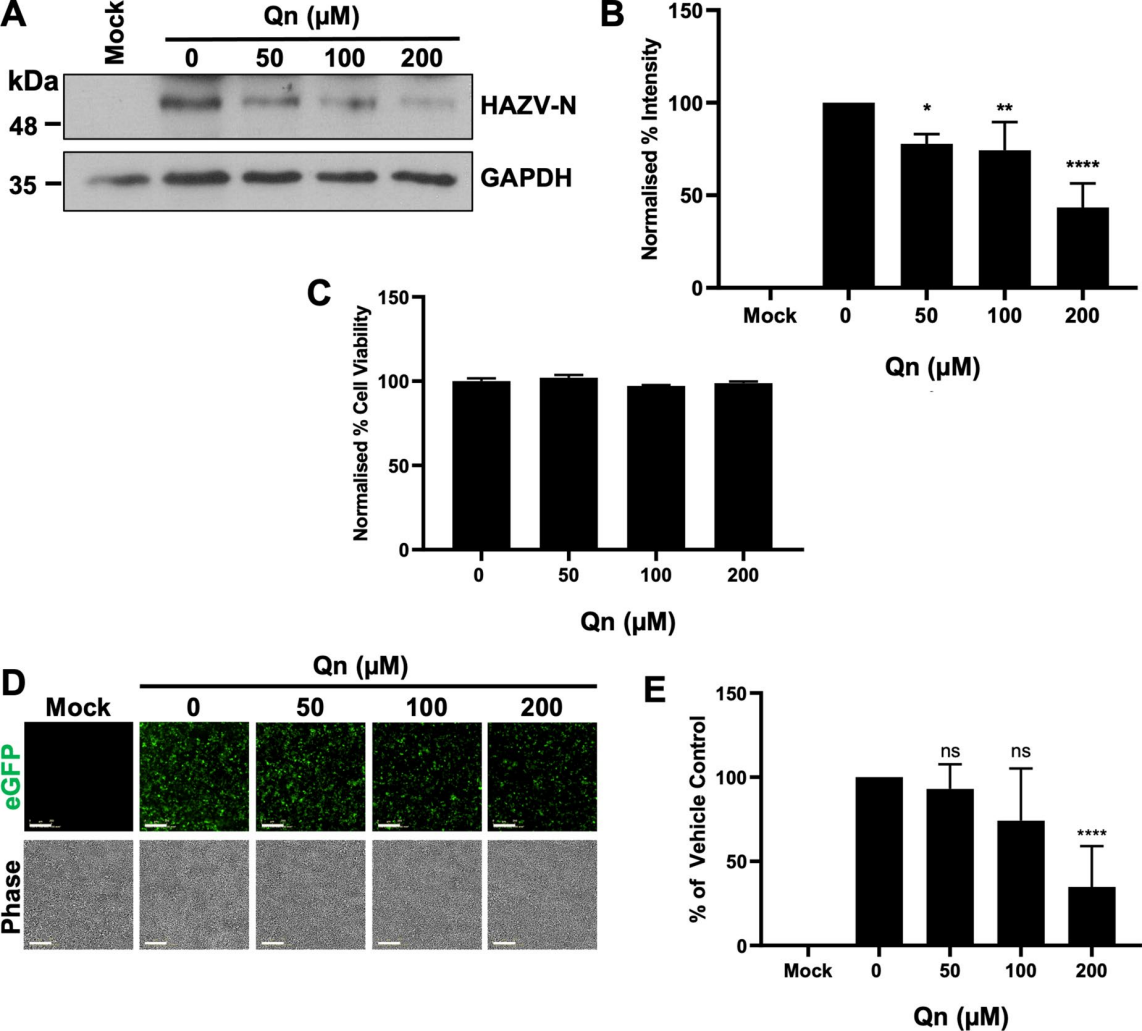
HAZV is sensitive to blockade of K^+^ channels. (**A**) A549 cells were pre-treated with the indicated concentrations of Qn or a DMSO control (0 µM) for 45 min. Cells were then infected with rHAZV-wt (MOI 0.1) in the presence of drug. Cells were lysed 24 hpi and lysates were resolved by SDS-PAGE and probed for HAZV-N expression using sheep anti-HAZV-N polyclonal antibody (1:5000) and rabbit anti-goat secondary antibody (1:10,000) by western blot. GAPDH was used as a loading control. (**B**) Densitometry analysis of (**A**). Band densities were calculated and normalised to untreated (0 µM) cells. *n* = 4. (**C**) To test for cell viability, cells were maintained in drug-containing medium for 24 h at 37°C and cell viability assessed by MTS assay. Cell viability was normalised to cells treated with a vehicle control and error bars represent ± S.D. Toxicity assays performed across 3 × technical repeats. (**D**) A549 cells were pre-treated with Qn or a vehicle control as in (**A**). Cells were infected with rHAZV-eGFP and imaged at 24 hpi using the IncuCyte Zoom system (scale bar = 200 µm). Representative images are shown. (**E**) Quantification of (**D**). Total integrated intensity of eGFP (TIIE) was calculated for each image set and normalised to untreated cells (0 µM). *n* = *8.* The statistical significance of both densitometry and eGFP results were determined by one-way ANOVA followed by Dunnett’s multiple comparisons test versus control (0 µM); ns, *p* > 0.05; *, *p* < 0.05; **, *p* < 0.01; ****, *p* < 0.001. Error bars represent standard deviation.

The siRNA screen revealed the most abundant class of cellular channels affecting HAZV activities were K^+^ channels, with almost half of the top 25 channel hits being from this group. To further confirm the role of K^+^ channels in HAZV multiplication, we next tested whether broad-spectrum K^+^ channel inhibitors Qn (Fig. 2), Qd and tetra-ethylammonium (TEA) (Supplementary Fig. 4) impeded HAZV activities, with each of these inhibitors known to impede the function of multiple K^+^ channels^24–26^.

In cells pre-treated with Qn (Fig. 2), which is clinically approved to treat malaria, quantification of western blots measuring HAZV-N expression at 24 hpi showed Qn inhibited HAZV gene expression in a concentration-dependent manner (Fig. 2A and B), at non-toxic concentrations of 50 µM, 100 µM and 200 µM (Fig. 2C) reducing HAZV-N abundance by 22.8%, 25.6% and 56.6%, respectively. The efficacy of these compounds was confirmed using rHAZV-eGFP, where pre-treatment of A549 cells with 50 µM, 100 µM and 200 µM reduced eGFP TIIE by 7%, 25.9% and 65.2%, respectively (Fig. 2D and E). A similar concentration-dependent effect on rHAZV gene expression was seen for Qd (Supplementary Fig. 4A–D), a clinically approved antiarrhythmic agent, and TEA (Supplementary Fig. 4E–H), at corresponding non-toxic concentrations (Supplementary Fig. 4I and J), as previously reported^12^. These findings corroborate the siRNA screen data revealing involvement of cellular K^+^ channel function in HAZV multiplication.

### HAZV is most sensitive to K^+^ channel blockade early in the viral life cycle

**Fig. 3:**
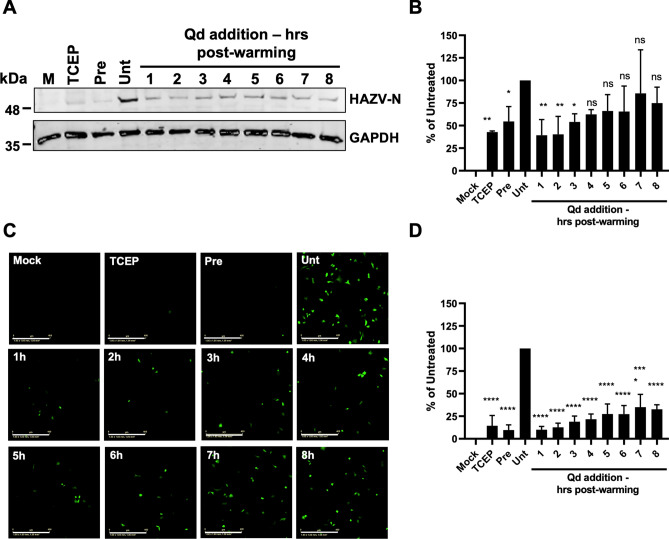
Qd impedes HAZV infection at an early stage of the life cycle. (**A**) rHAZV-wt was bound to A549 cells in serum-free medium (MOI 0.1) at 4°C for 1 h. Inoculum was removed and cells were washed with PBS. Complete DMEM was pre-warmed to 37°C and added to cells to initiate internalisation (t = 0). At the time points indicated, DMEM was removed and 200 µM Qd (or DMSO, 0 µM, ‘Unt’) was added to cells. One well was treated with 10 mM TCEP prior to warming as a control for early internalisation. One was treated with Qd before and during infection as a control for drug activity (Pre). Cell lysates were collected at 24 h post-warming and resolved by SDS-PAGE. HAZV-N expression was detected by western blot, with GAPDH used as a loading control. (**B**) Densitometry analysis of (**A**). Band intensity was normalised to untreated cells (Unt). *n* = 4. (**C**) rHAZV-eGFP was bound to A549 cells at 4°C and time of addition assays were carried out as in (**A**). Images of infected cells were captured using the IncuCyte S3 (scale bar = 400 µm) at 24 h post-warming. (**D**) Quantification of (**C**) as in Fig. 2, expressed as a percentage of untreated cells (Unt). *n* = *7*. The statistical significance of both densitometry and eGFP results were determined by one-way ANOVA followed by Dunnett’s multiple comparisons test versus control (Untreated); ns, *p* > 0.05; *, *p* < 0.05; **, *p* < 0.01; ***, *p* < 0.005; ****, *p* < 0.001. Error bars represent standard deviation.

The broad-spectrum K^+^ channel blocker assays used in the previous section measured translation of virus-encoded N protein or eGFP over 24 h as markers for virus multiplication. As this window of measurement encompassed at least one round of infection, it was possible that reduced N protein or eGFP expression in response to drug treatment involved disruption at one or more stage of the replication cycle, including virus entry, gene expression, assembly and egress. To better characterise how K^+^ channel blockade influenced HAZV infection, we performed time-of-addition assays, similar to our previous work involving other bunyaviruses^27^. We bound rHAZV-wt or rHAZV-eGFP to A549 cells at 4 °C, preventing virus internalisation, with synchronization of viral entry achieved by subsequent warming to 37°C. Cells were then treated with 200 µM Qd, which we have previously shown to effectively inhibit HAZV multiplication (Supplementary Fig. 4A–D and Punch et al.^12^), at hourly time points for 8 h post-warming. One well was washed with 10 mM TCEP immediately post-warming as a control for internalisation/entry during the initial binding steps, as TCEP effectively reduces disulfide bonds in proteins^28^, thus rendering viral glycoproteins unable to facilitate fusion. Western blot analysis of cell lysates harvested at 24 hpi (Fig. 3A and B) demonstrated that the most significant inhibition of rHAZV-wt infection occurred when added between 1 and 3 h post-warming, with levels of N expression similar to that of pre-infection treated positive control cells. For rHAZV-eGFP, inhibition was again greatest within the first 4 h post-warming, with a plateau after this time (Fig. 3C and D). This trend matched that observed for NH_4_Cl treatment (Supplementary Fig. 5), which is a known inhibitor of endosome acidification, and blocker of virus entry^29^. Therefore, this suggests that broad-spectrum K^+^ channel blockade acts upon the entry stage of the HAZV life cycle.

### The clinically approved K_V_ blocker dronedarone inhibits HAZV infection at low micromolar concentrations

**Fig. 4:**
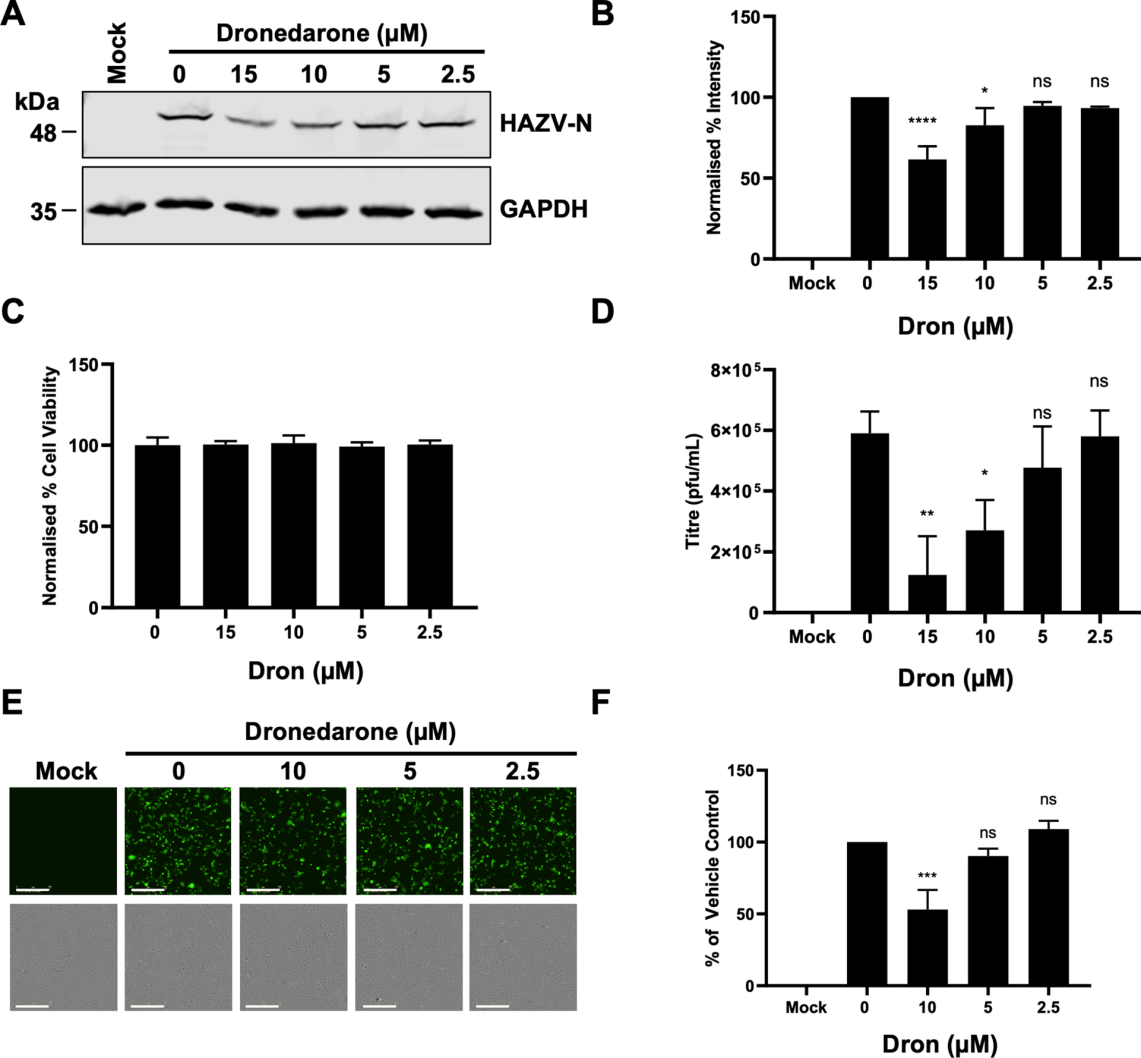
The clinically approved K^+^ channel blocker dronedarone inhibits HAZV infection at low micromolar concentrations. (**A**) A549 cells were pre-treated with the indicated concentrations of dronedarone or a DMSO control (0 µM) for 45 min. Cells were then infected with rHAZV-wt (MOI 0.1) in the presence of drug. Cells lysates were collected at 24 hpi and resolved by SDS-PAGE. HAZV-N expression was detected by western blot, with GAPDH used as a loading control. (**B**) Densitometry analysis of (**A**). Band densities were calculated and normalised to untreated (0 µM) cells. *n* = 3. (**C**) To test for cell viability, cells were maintained in drug-containing medium for 24 h at 37 °C and cell viability assessed by MTS assay with 3 × technical repeats. Cell viability was normalised to cells treated with a vehicle control, with error bars representing ± S.D. (**D**) A549 cells were pre-treated with dronedarone or a vehicle control as in (**A**). Cell supernatants were harvested 24 hpi and released virus was quantified by plaque assay on SW13 cells. *n* = 3. (**E**) Cells were pre-treated with Dron or a vehicle control as in (**A**) and infected with rHAZV-eGFP and imaged 24 hpi using the IncuCyte Zoom system (scale bar = 200 µm). Representative images are shown. (**F**) Quantification of (**E**) as in Fig. 2, normalised to untreated cells (0 µM). *n* = 3. The statistical significance of densitometry, plaque assay and eGFP results were determined by one-way ANOVA followed by Dunnett’s multiple comparisons test versus control (0 µM); ns, *p* > 0.05; *, *p* < 0.05; **, *p* < 0.01; ***, *p* < 0.005; ****, *p* < 0.001. Error bars represent standard deviation.

Following siRNA screening for cellular ion channels that support HAZV infection and the subsequent testing of broad-spectrum pharmacological K^+^ channel blockers, we sought to test the efficacy of more specific K^+^ channel inhibitors for their anti-HAZV activity. To this end, we investigated the anti-HAZV activity of the compound Dron, an FDA-approved drug for the treatment of heart arrythmias that targets members of the K_V_ and K_2P_ families^30,31^, of which 24 members were covered in our siRNA screen and 6 appeared amongst the top 25 hits (Fig. 1C). Quantification of western blots (Fig. 4A and B) revealed a concentration-dependent inhibition of rHAZV-wt infection at low micromolar doses, with 17.3 and 28.5% inhibition in cells pre-treated with non-toxic 10 µM and 15 µM dronedarone concentrations (Fig. 4C), respectively. Quantification of virus released into cell supernatants demonstrated that pre-treatment of cells with 10 µM and 15 µM dronedarone reduced virus release by 54.7% and 78.6%, respectively (Fig. 4D). Corroborating these results, quantification of cells pre-treated with 10 µM dronedarone and infected with rHAZV-eGFP showed a reduction in TIIE of 46.8% (Fig. 4E and F). These results suggest dronedarone is a promising anti-HAZV compound.

### Exposure of HAZV to K^+^ delays acid-mediated inactivation of virus infectivity

**Fig. 5:**
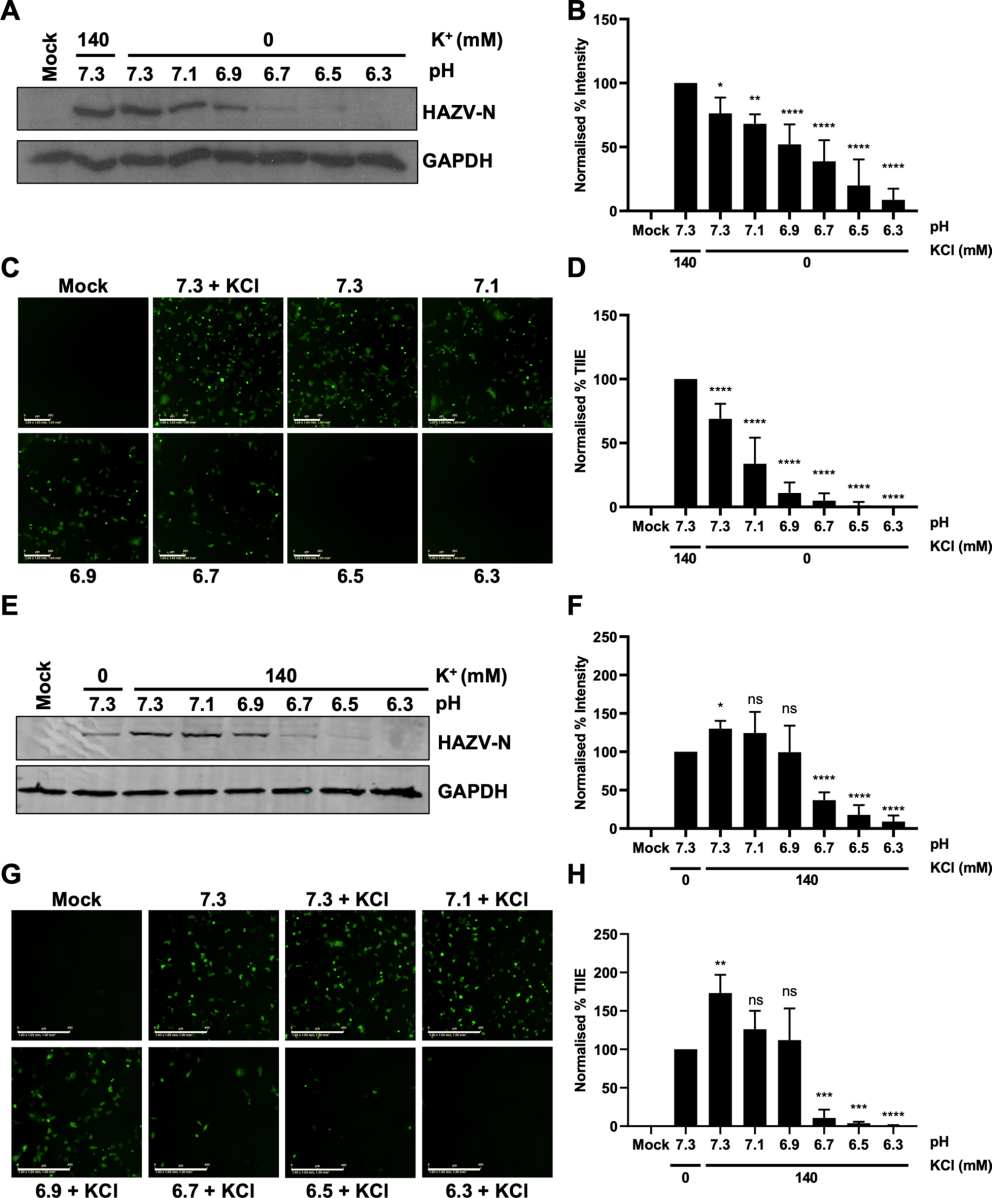
K^+^ exposure protects HAZV from acid-mediated inactivation until pH 6.9. (**A**) rHAZV-wt was incubated in buffers maintained at pH 7.3 ± 140 mM KCl, 7.1, 6.9, 6.7, 6.5 or 6.3 without any additional salt for 2 h. Virus and buffer mix was diluted 1:11 in DMEM and added to cells (MOI 0.1). Cells were then lysed at 18 hpi and lysates were resolved by SDS-PAGE. HAZV-N expression was detected by western blot, with GAPDH used as a loading control. (**B**) Densitometry analysis of (**A**), normalised to pH 7.3 + 140 mM KCl. *n* = 4. (**C**) rHAZV-eGFP was pre-treated with the indicated buffers in (**A**) for 2 h. Cells were infected as in (**A**) and imaged at 18 hpi (scale bars = 400 μm) using an IncuCyte Zoom system. (**D**) Quantification of (**C**) as in Fig. 2, normalised to pH 7.3 + 140 mM KCl treatment. *n* = 4. (**E**) rHAZV-wt was incubated in buffers maintained at pH 7.3 ± 140 mM KCl, or 7.1, 6.9, 6.7, 6.5 and 6.3 + 140 mM KCl for 2 h. Virus and buffer mix was diluted 1:11 in DMEM and added to A549 cells at MOI 0.1. Cells were lysed at 18 hpi and processed as in (**A**). (**F**) Densitometry analysis of (**E**) normalised to pH 7.3 + 0 mM KCl. *n* = *7.* (**G**) rHAZV-eGFP was pre-treated with the indicated buffers as in in (**E**) for 2 h. Cells were infected and imaged at 18 hpi (scale bars = 400 μm) using an IncuCyte Zoom system. (**H**) Quantification of (**G**), normalised to pH 7.3 + 0 mM KCl. *n* = *3*. The statistical significance of both densitometry and eGFP results were determined by one-way ANOVA followed by Dunnett’s multiple comparisons test versus control (pH 7.3 + 140 mM for (**B**) and (**D**); pH 7.3 + 0 mM KCl for (**E**) and (**G**); ns, *p* > 0.05; *, *p* < 0.05; **, *p* < 0.01; ***, *p* < 0.005; ****, *p* < 0.001. Error bars represent standard deviation.

The results described above, alongside our work on HAZV spike structure^12^, show a role for K^+^ during HAZV infection, specifically during virus entry. Considering that pH and K^+^ concentration are in flux within native maturing endosomes^14^, we next characterised the combined effect of both H^+^ and K^+^ on rHAZV-wt infection using an in vitro assay. To this end we exposed viruses to defined buffer conditions for which the pH dropped by increments of 0.2 pH units, which was diluted out prior to addition to cells, as described elsewhere^12,27,32,33^ (Fig. 5A–D). Our similar work with Bunyamwera virus (BUNV) has demonstrated that exposure of cell surface-bound virions to the pH around that of fusion in the presence of K^+^ can induce fusion at the plasma membrane^33^, suggesting that these conditions maintain a fusion-ready state within the glycoprotein spikes and thus enhancing the likelihood of successful infection. Here, in the absence of K^+^, pre-incubation of rHAZV-wt in buffers of decreasing pH led to a stepwise decrease in rHAZV-wt infection, as measured by HAZV-N detection by western blot (Fig. 5A and B). Each incremental drop in pH, even between pH values of 7.3–7.1, resulted in a significant drop in HAZV-N abundance, suggesting that HAZV virions tolerate a very narrow range of pH conditions that permit maximal infection. In agreement with previous work^12^, the results also showed an almost complete loss of infection at pH 6.3. A similar outcome was also observed for rHAZV-eGFP under identical conditions (Fig. 5C and D), with TIIE decreasing significantly in a stepwise manner alongside decreasing pH, with infection abrogated at pH 6.3. These results suggest that any reduction in pH below 7.3 in the absence of K^+^ leads to a progressive reduction in virion infectivity.

Next, we repeated this infection assay with the addition of K^+^ across all buffer conditions. As in our previous work^12^, the addition of K^+^ to pH 7.3 buffers significantly increased rHAZV-wt infection (Fig. 5E and F). However, in contrast to when K^+^ was omitted (Fig. 5A and B), the addition of K^+^ to pH 7.1 and pH 6.9 buffers resulted in no significant loss in HAZV-N detection (Fig. 5E and F). A similar finding was also observed when rHAZV-eGFP was subjected to the same assay, where addition of K^+^ resulted allowed maximal infectivity across pH values from 7.3 to 6.9 (Fig. 5G and H vs Fig. 5C and D). Importantly, HAZV infection was also expedited when treated at concentrations ranging from 20 to 140 mM (Supplementary Fig. 6), which are within the range that is generally accepted to be found at early endosomes^14^. These results suggest that in the presence of elevated K^+^ concentrations, a physiological condition found in endosomes, HAZV virions are more resistant to low pH, potentially increasing the window of opportunity for virions to fuse and enter the host cell while transiting through the endolysosomal network.

## Discussion

The internalisation pathway(s) of bunyaviruses are not fully characterised. Common features between the entry requirements of members of the *Peribunyaviridae, Phenuiviridae, Nairoviridae* and *Hantaviridae* families are thought to exist, with members of each of these families having demonstrated dependencies during endocytic internalisation^34–37^. As endosomes mature, their intralumenal conditions change through acidification and ion flux across the endosomal membrane, facilitated by ion channel activity^14^. There is growing evidence that flux of various ions such as K^+^ represents a critical co-factor in viral entry from endosomes, alongside decreasing pH^13^. Therefore, we sought to identify the cellular ion channels that may be involved in HAZV entry. To this end, we carried out an siRNA screen targeting ion channel-encoding genes. Amongst our hits, multiple voltage-gated (K_V_) and two-pore (K_2P_) K^+^ channels were identified as pro-viral factors, as well as several Ca^2+^ and non-selective cation channels (Fig. 1). Of these hits, members of the *KCNN* (K_2P_) subfamily have previously been identified as pro-viral factors during entry of the peribunyavirus BUNV using pharmacological screening^32^. Additionally, the top 3 hits in a similar siRNA-based ion channel screen for lymphocytic choriomeningitis arenavirus (LCMV), namely KCND2, KCNJ13 and KCNN3^16^ were also hits for HAZV (Fig. 1). Therefore, these results suggest a bunyavirus-wide requirement of cellular K^+^ channels, even if the mechanism of action seems to differ across families: while K^+^ is involved in conformational changes of the viral glycoproteins during fusion for orthobunyaviruses^33^ and nairoviruses^12^, it is involved in uncoating for arenaviruses^16^.

The proposition of repurposing ion channel inhibitors as antiviral therapies has received increasing attention over the last decade^13,38,39^. Therefore, after finding that HAZV infection is sensitive to genetic knockdown of several K^+^ channels, we sought to verify these observations with broad-spectrum pharmacological blockade (Fig. 2). Previous work has shown that Qd and TEA inhibit HAZV infection^12^, so first we confirmed this finding for rHAZV-wt and rHAZV-eGFP (Supplementary Fig. 4). Next, we tested other channel blockers for their ability to inhibit HAZV infection. Using the broad-spectrum K^+^ channel blocker Qn, we demonstrated a concentration-dependent inhibition of HAZV gene expression following pre-treatment of cells. Our results for Qn corroborate similar findings for BUNV^27^ and LCMV^16^, and demonstrate a similar concentration-dependent effect seen when using Qd against HAZV. Following this, we examined the temporal inhibition profile of Qd-mediated K^+^ channel blockade, to identify at which stage of the viral replication cycle K^+^ was involved (Fig. 3). Previous work has implied this stage to be entry, and more specifically endosome escape, due to a direct effect of K^+^ on conformational and fusogenic properties of the glycoprotein spikes. Time-of-addition assays with Qd demonstrated highest inhibition of HAZV gene expression when added to cells soon after attachment to cells, within 1–3 h post-binding. This inhibition profile matched that of NH_4_Cl (Supplementary Fig. 5), known to block virus envelope fusion within endosomes, suggesting that K^+^ channel activity is required for HAZV endosome escape.

Although Qd is clinically-approved for treating arrythmia^40^, we sought to assay a K^+^ channel blocker with narrower activity spectra, to corroborate our siRNA screen results. Thus, we next used the clinically-approved anti-arrhythmic agent dronedarone^30,31^ and showed it to inhibit HAZV activity within the low micromolar range, with a significant drop in released virus titres (Fig. 4).

Finally, we sought to better understand the coordinated effect of K^+^ and low pH during HAZV entry using a ‘priming’ assay. By incubating virions in buffers of decreasing pH, we showed a pH-dependent loss of infectivity, likely through acid-mediated conformational changes in the Gn/Gc architecture to a post-fusion conformation and thus inactivation. Interestingly, addition of K^+^ to pH buffers altered the pH threshold at which viruses are inactivated (Fig. 5). We hypothesise that K^+^ may stabilize viral spikes in an intermediate conformation for longer, potentially the one observed when HAZV virions were exposed to K^+^^12^, delaying acid-mediated switching to a post-fusion conformation and increasing chances of engagement with target membranes in acidifying compartments. Assays using more physiological concentrations, i.e. 20 and 60 mM, as proposed for early and late endosomes respectively^14^, also demonstrated an enhancing effect on infection at pH 7.3 (Supplementary Fig. 6), suggesting that our results are not a result of excess KCl.

Taken together, our findings extend our knowledge of nairovirus entry, identifying a role for several K^+^ channels as pro-viral factors during early stages of the HAZV life cycle, which are druggable targets. In light of the common features of HAZV and CCHFV entry mechanisms, it would be exciting to investigate the susceptibility of CCHFV to K^+^ channel blockade and assess whether this may be a viable antiviral strategy.

## Methods

### Cells and viruses

A549 (human epithelial alveolar carcinoma) and SW13 (human adrenal carcinoma) cells were obtained from the American Type Culture Collection (ATCC) and maintained in a humidified incubator at 37°C with 5% CO_2_. Cells were cultured in high-glucose Dulbecco’s modified Eagle’s medium (DMEM, Sigma-Aldrich) supplemented with 10% foetal bovine serum (FBS), 100 U/mL penicillin and 100 μg/mL streptomycin (final concentration; 1% pen/strep); termed ‘complete’ DMEM herein. BSR/T7 (baby hamster kidney, stably expressing bacteriophage T7 RNA polymerase) cells were cultured in complete DMEM and selected with 500 μg/mL G418 every two passages.

Recombinant HAZV (rHAZV-wt) and rHAZV expressing eGFP as a non-fused reporter protein (rHAZV-eGFP) have been described previously^15,41^. Briefly, BSR/T7 cells were seeded onto 6 well plates (~ 3 × 10^5^ cells/well) in complete DMEM and allowed to grow to 70–80% confluence. Cells were transfected with a transfection mixture containing 1.2 μg pMK-RQ-S-wt or -eGFP, pMK-RQ-M, and pMK-RQ-L; 0.6 μg pCAG-T7pol (Addgene plasmid number 59926); 2.5 μL JetOptimus (Polyplus) transfection reagent per microgram of DNA and 200 μL JetOptimus transfection buffer. The transfection mixture was incubated at room temperature for 15 min to complex cDNAs and transfection reagent and added dropwise to cells. At 8 h post-transfection (hpt) the transfection mixture and media were removed and replaced with fresh DMEM supplemented with 2.5% FBS and 1% pen/strep. Supernatants were harvested at 120 hpt and clarified by centrifugation at 1500 x *g*. Recombinant viruses were bulked by inoculating SW13 cells with virus-containing supernatant for 120 h, followed by further clarification and storage of aliquots at − 80°C.

### Quantification of virus stocks by plaque assay

SW13 cells were seeded onto 12-well plates (1 × 10^5^ cells/well) and allowed to grow to 80% confluency. HAZV supernatants were tenfold serially diluted. Each dilution was added onto cells in duplicate wells and allowed to adsorb for 1 h at 37°C, followed by overlay with 1.6% w/v carboxymethyl cellulose (CMC) mixed 1:1 with complete DMEM. Cells were incubated for 6 days and fixed with 4% paraformaldehyde (PFA) by direct addition on top of the CMC/DMEM overlay for 1 h at 4°C. Cells were washed twice with dH_2_O and stained with 1% crystal violet solution (Sigma) for 15 min. Cells were washed two more times with dH_2_O and the number of plaques was counted to calculate the viral titre. All raw virus titre data are included in Supplementary data set 2.

### Cell viability assays

A549 cells were seeded into 96-well plates and pre-treated with the specified inhibitors for 1 h and incubated at 37°C for 24 h. At 24 h post- treatment, 20 μL CellTiter 96 MTS reagent (Promega) was added to each well and cells were incubated in the dark at 37°C for 4 h. Cell viability was measured by reading absorbance at 492 nm using a Magellan F50 plate reader. All raw viability data are included in Supplementary data set 2.

### Drug treatment assays

A549 cells were pre-incubated for 45 min at 37°C in complete DMEM supplemented with the indicated ion channel inhibitor or a vehicle control for each inhibitor (procainamide, disopyramide, nifedipine, quinidine and quinine were prepared as 100 mM stocks in dimethyl sulfoxide (DMSO); tetrandrine and dronedarone were prepared as a 5 mM stock in DMSO; lidocaine was prepared as a 1 mM stock in DMSO; TEA and NH_4_Cl were prepared as 1 M stocks in water). rHAZV-wt or rHAZV-eGFP was added directly to wells in the presence of drug (MOI 0.1) and cells were incubated for 24 h at 37°C. Cells were lysed (rHAZV-wt) or live-imaged (rHAZV-eGFP) 24 hpi for western blot or fluorescence imaging, respectively. For virus release assays, supernatant was harvested 24 h post-infection and clarified by centrifugation at 17,000 × *g* for 5 min. Infectious virus in harvested supernatant was quantified by plaque assay in SW13 cells.

### Time of addition assays

rHAZV-wt or rHAZV-eGFP was bound to A549 cells in cold serum-free medium at 4°C for 1 h. Cells were warmed to 37°C by replacing infection media with complete DMEM pre-warmed to 37°C. Cells were treated with 20 mM NH_4_Cl or 200 μM Qd at the indicated time-points post-warming. One well was washed with 10 mM TCEP for five minutes at t = 0 as an internalisation control. Cells were assayed for infection via western blotting or fluorescent imaging for rHAZV-wt and rHAZV-eGFP respectively. One well was washed with PBS and treated with 10 mM cold tris-carboxyethyl phosphine (TCEP) in serum-free media immediately post-warming to control for internalisation during the binding stage.

### Fluorescence imaging—eGFP readout

rHAZV-infected cells were imaged using the IncuCyte S3 live cell imaging system (Sartorius) with a 10 × objective. Wide-field images of 2.15 mm^2^ were taken to identify infected cells and the total integrated intensity of the corresponding fluorescent signals was quantified and normalised to controls. For time-course analysis, A549 cells infected with rHAZV-eGFP were live-imaged using the IncuCyte S3 over a period of 8 h. Total integrated intensity of green fluorescence signal was calculated and processed as above. All raw IncuCyte data are included in Supplementary data set 2.

### Western blotting

At the time points indicated, cells were washed with 1 × PBS and lysed using lysis buffer (25 mM glycerol phosphate, 20 mM tris, 150 mM NaCl, 1 mM EDTA, 1% triton, 10% glycerol, 50 mM NaF, 5 mM Na_4_O_7_P_2_, pH 7.4) supplemented with HALT protease inhibitor cocktail (Thermo Scientific). Cells were lysed for 15 min with gentle rocking at 4°C and lysates were collected using a cell scraper. Lysates were clarified by centrifugation at 17,000 × *g* for 10 min and stored at −20°C. After SDS-PAGE and transfer onto methanol-activated polyvinylidene difluoride (PVDF, Millipore) membranes, western blot was performed labelling proteins with primary antibodies followed by the corresponding IRDye secondary antibodies (Licor). Labelling was detected using the Licor Odyssey Sa Infrared imaging system. Densitometry analysis was performed over at least three independent experiments using ImageJ (NIH)^42^. All raw densitometry data are included in Supplementary data set 2.

### siRNA screen

siRNA screen was carried out in a protocol adapted from Shaw et al.^2^. The Silencer Human Ion Channel siRNA library (Thermo) was prepared prior to use by reconstituting 0.25 nmol of each siRNA in 125 μL nuclease-free H_2_O and mixing to generate a stock parental plate of 2 μM. Working stocks were prepared in duplicate by combining 50 μL per well from the stock parental plate with 50 μL nuclease-free water to generate a working plate at 1 μM (1 pmol/μL). For siRNA screening, a transfection master mix was made up containing 0.3 μL Lipofectamine RNAiMAX transfection reagent and 16.7 μL Opti-MEM per well. 17 μL of master mix was added to each well of a 96-well plate minus two for virus-only controls. 3 μL of 1 μM working stock siRNA was added to each well (final 3 pmol siRNA/well) and allowed to complex with the transfection mix for 15 min at room temperature. Trypsinised A549 cells were resuspended in complete DMEM, cell concentration was adjusted to 1.25 × 10^5^ cells/mL and 100 μL of cell suspension was added to each well (final 1.25 × 10^4^ cells/well). Cells were incubated with the transfection mixture for 8 h at 37°C, at which point 100 μL media was removed and replaced with 100 μL fresh complete DMEM. At 48 hpt, cells were infected with rHAZV-eGFP (MOI 0.1) and incubated for 24 h at 37 °C. Cells were live imaged using the IncuCyte S3 fluorescence imaging system. All raw IncuCyte data for the siRNA screen are included in Supplementary data set 1.

### In vitro acid/K + treatment assays

rHAZV-wt or rHAZV-eGFP was incubated at a ratio of 1:11 in the specified buffer(s) at 37°C for 2 h. 1 part virus/buffer mix was diluted in 10 parts complete DMEM to neutralise the pH and dilute the high salt concentration. The mixture was added to cells and infection was assayed after 18 h (unless otherwise stated) via western blotting or fluorescent imaging for rHAZV-wt and rHAZV-eGFP, respectively. Buffers were prepared on the day of use containing 20 mM tris for pH 6.7–7.3 buffers and 30 mM bis–tris for pH 6.3–6.5 buffers. K^+^ was supplemented using the salt KCl, as previous, to achieve the indicated final concentrations. Buffers were warmed to 37°C and pH was adjusted using 32% HCl immediately prior to use. Buffer pH was checked after the 2 h priming period to ensure maintenance of pH during the experiment.

#### Statistical analysis

Statistical significance of densitometry, eGFP and plaque assay data was analysed using one-way ANOVA followed by Dunnett’s multiple comparisons tests versus the respective controls in GraphPad Prism. Results were considered statistically significant when *p* < 0.05.

## Supplementary Information

Supplementary Information 1.

Supplementary Information 2.

Supplementary Information 3.

## Data Availability

All data generated or analysed during this study are included in this published article (and its Supplementary Information files).
