# Supplementary material for: Identification of cellular ion channels that facilitate Hazara nairovirus infection enables selection of clinically approved compounds with anti-nairoviral properties
Source: Sci Rep. 2026 Mar 24;16:14840. doi: 10.1038/s41598-026-42810-7 (PMC13168314; doi:10.1038/s41598-026-42810-7)
Supplement: Supplementary file 3 — Supplementary Information 3. [file 41598_2026_42810_MOESM3_ESM.pdf]

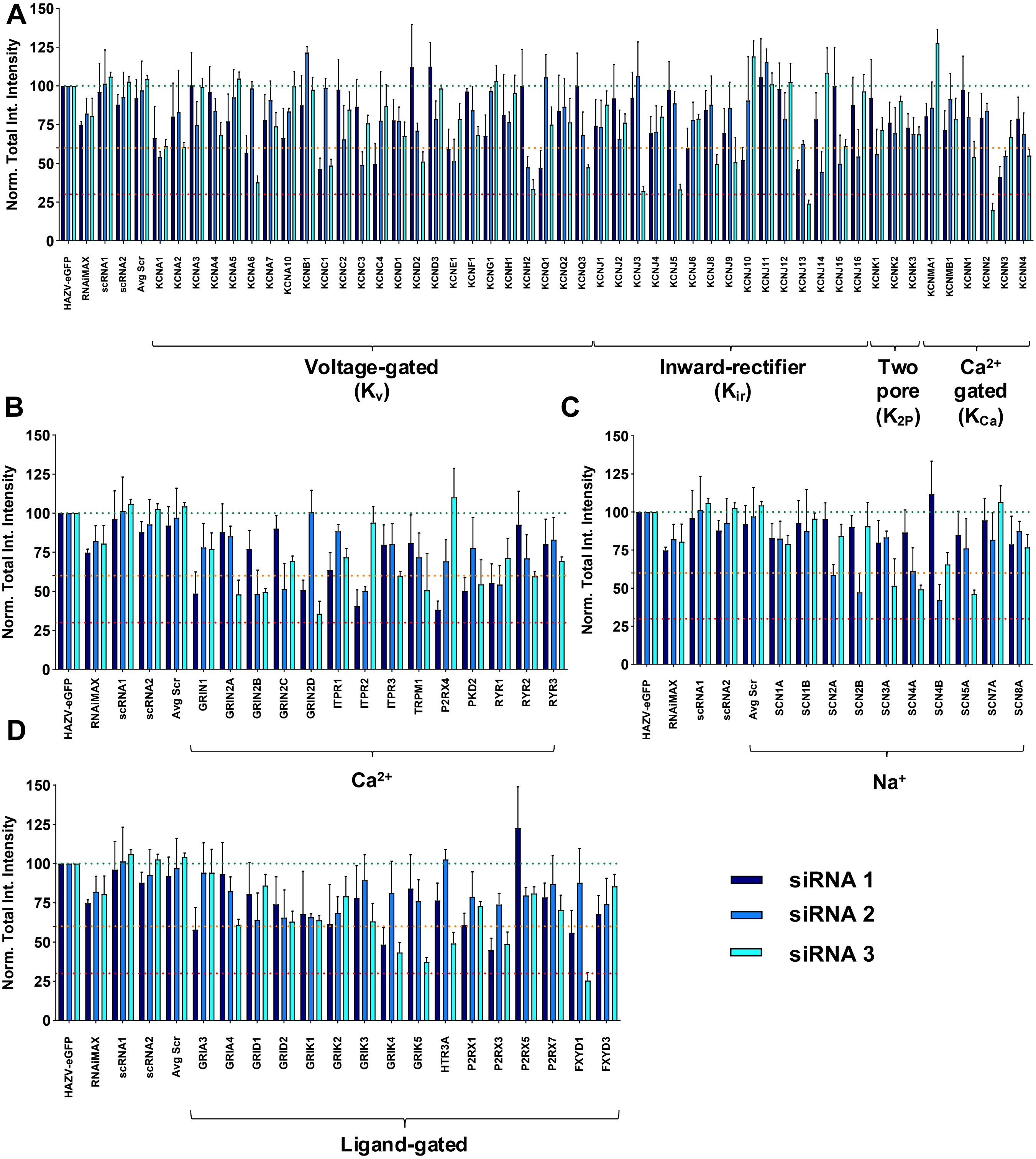

Supplementary fig. 1. Normalized eGFP intensity values for each individual siRNA used in the ion channel screen. Individual results for each siRNA for the screen described in Figure 1. Average TIIE in transfected A549 cells infected with rHAZV-eGFP 24 hpi. Bars represent the average of two repeats of one of three siRNAs targeting each gene: (siRNA 1 = dark blue, siRNA 2 = medium blue, siRNA 3 = light blue). Scrambled RNA (scRNA 1 and 2) and transfection reagent plus HAZV-eGFP (RNAiMAX) controls are also shown alongside HAZV-eGFP-infected cells. Red, orange and green lines mark the 30th, 60th and 100th percentile, respectively. (A) Histograms of siRNAs targeting K^+ channels, grouped by gating. (B) Histograms of siRNAs targeting Ca^{2+} channels. (C) Histograms of siRNAs targeting Na^+ channels. (D) Histograms of siRNAs targeting ligand-gated channels.

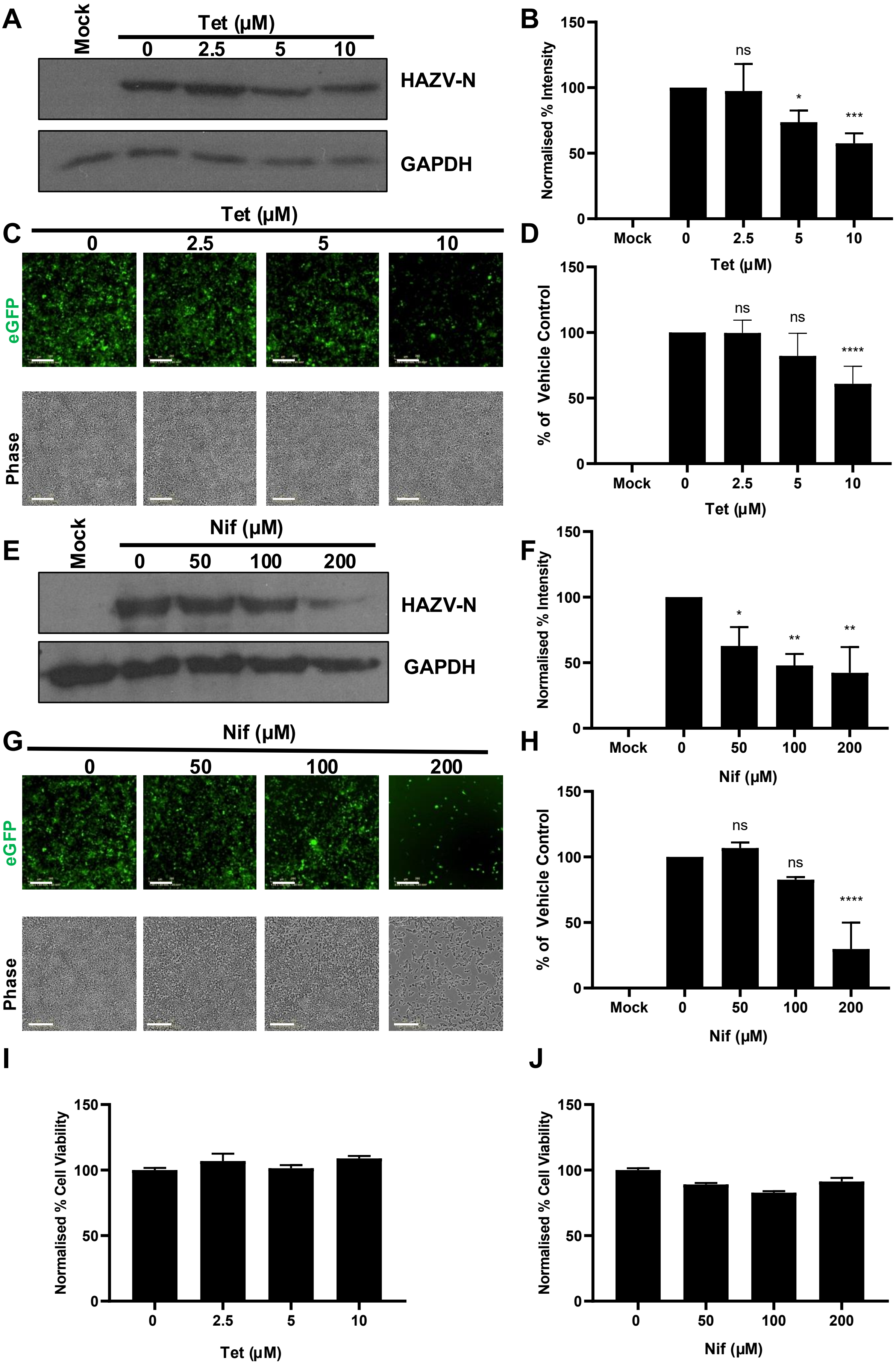

A
Mock
Tet (μM)
0
2.5
5
10
HAZV-N
GAPDH
B
Normalised % Intensity
150
100
50
0
ns
*
***
Mock
0
2.5
5
10
Tet (μM)
C
Tet (μM)
0
2.5
5
10
eGFP
Phase
D
% of Vehicle Control
150
100
50
0
ns
ns
****
Mock
0
2.5
5
10
Tet (μM)
E
Mock
Nif (μM)
0
50
100
200
HAZV-N
GAPDH
F
Normalised % Intensity
150
100
50
0
*
**
**
Mock
0
50
100
200
Nif (μM)
G
Nif (μM)
0
50
100
200
eGFP
Phase
H
% of Vehicle Control
150
100
50
0
ns
ns
****
Mock
0
50
100
200
Nif (μM)
I
Normalised % Cell Viability
150
100
50
0
0
2.5
5
10
Tet (μM)
J
Normalised % Cell Viability
150
100
50
0
0
50
100
200
Nif (μM)

Supplementary fig. 2 – HAZV is sensitive to blockade of Ca^{2+} channels . (A) A549 cells were pre-treated with the indicated concentrations of Tet or a vehicle control (0 µM) for 45 minutes. Cells were infected with rHAZV-wt (MOI 0.1 in the presence of drug). Cells were lysed 24 hpi and lysates were resolved by SDS-PAGE and probed for HAZV-N expression using sheep anti-HAZV-N monoclonal antibody (1:5000) and rabbit anti-goat secondary antibody (1:10,000). GAPDH was used as a loading control. (B) Densitometry analysis of (A). Band densities were calculated and normalised to untreated (0 µM) cells. ns, p>0.05, **, p<0.01, ****, p<0.001. *n=6* (C) A549 cells were pre-treated with Tet or a vehicle control as in (A). Cells were infected with rHAZV-eGFP and imaged 24 hpi using the IncuCyte Zoom system (scale bar = 200 µm). Representative images are shown. (D) Quantification of (C). Total integrated intensity of eGFP (TIIE) was calculated for each image set and normalised to untreated cells (0 µM). ns, p>0.05, ****, p<0.001, *n=4*. (E) Infection assay as in (A) with the indicated concentrations of Nif. Cells were lysed and probed for HAZV-N by western blot as in (A). (F) Densitometry analysis of (E) as in (A). (G) Infection assay with rHAZV-eGFP as in (C) with the indicated concentrations of Nif. (H) TIIE quantification of (G) as in (D). To test for cell viability (I and J), cells were maintained in drug-containing medium for 24 hours at 37 °C and cell viability assessed by MTS assay with 3x technical repeats. Cell viability was normalised to cells treated with a vehicle control, with error bars represent ±S.D.

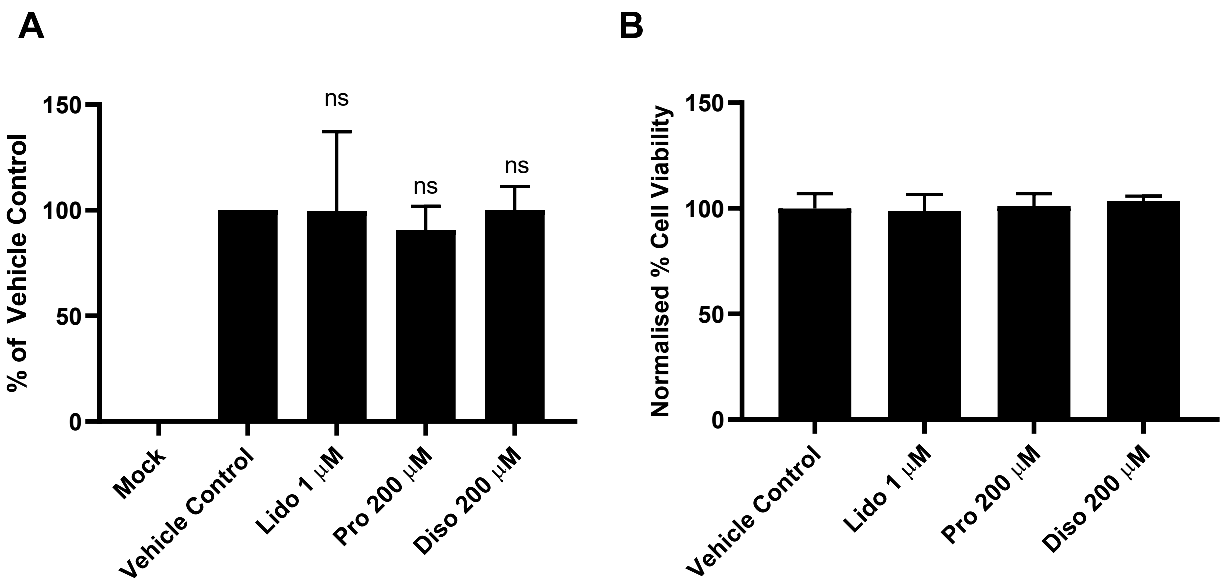

Supplementary fig. 3. HAZV infection is not sensitive to Na^+ channel blockade. A. A549 cells were pre-treated in triplicate with 1 µM lidocaine (Lido), 200 µM procainamide (Pro), 200 µM disopyramide (Diso) or a vehicle control for 45 minutes. Cells were infected with rHAZV-eGFP (MOI 0.1) for 24 hours. Whole well images were taken using the IncuCyte S3 and eGFP expression was measured as mean integrated green intensity. Mean integrated green intensity was normalised to the vehicle. ns, p>0.05, error bars represent ±S.D.; *n=3.* To test for cell viability (B), cells were maintained in drug-containing medium for 24 hours at 37 °C and cell viability assessed by MTS assay. Cell viability was normalised to cells treated with a vehicle control and error bars represent ±S.D.; n=3 for infection assays. 3x technical repeats for toxicity assays.

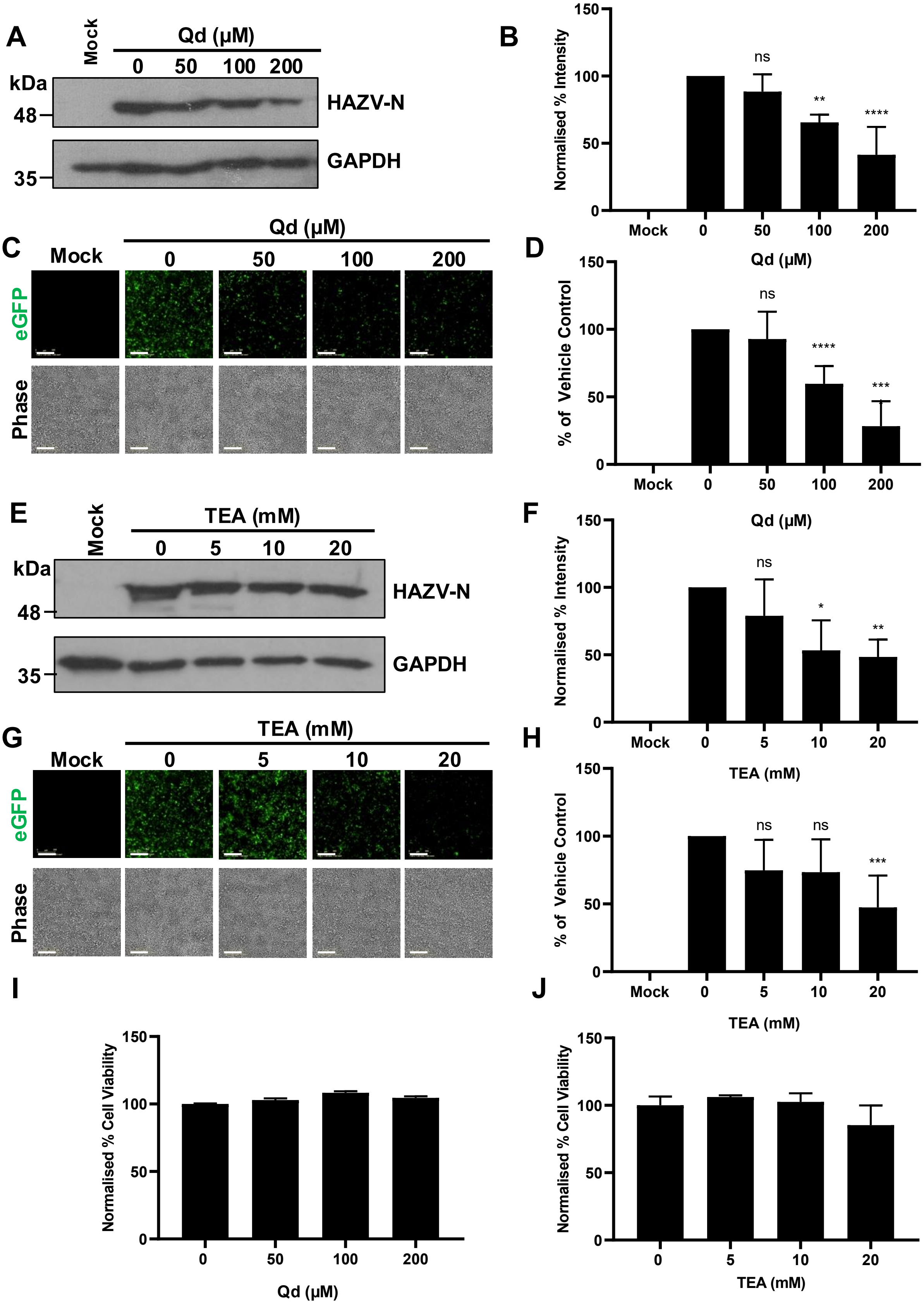

A
Mock
Qd (μM)
0 50 100 200
kDa
48
35
HAZV-N
GAPDH
B
Normalised % Intensity
ns
**
****
Mock 0 50 100 200
Qd (μM)
C
Mock
eGFP
Phase
D
% of Vehicle Control
ns
****
***
Qd (μM)
E
TEA (mM)
0 5 10 20
F
*
TEA (mM)
G
H
I
Normalised % Cell Viability
J

Supplementary fig. 4. HAZV is sensitive to blockade of K^+ channels. (A and E) A549 cells were pre-treated with the indicated concentrations of Qd (A) or TEA (E) or a vehicle control (DMSO and H_2O for Qd and TEA, respectively (0 µM, 0 mM)) for 45 minutes. Cells were then infected with rHAZV-wt (MOI 0.1) in the presence of drug. Cells were lysed 24 hpi and lysates were resolved by SDS-PAGE and resolved by SDS-PAGE. HAZV-N expression was detected by western blot, with 1:10000 GAPDH was used as a loading control. (B and F) Densitometry analysis of (A) and (E), normalised to untreated cells (0 µM). *n=4* for Qd; *n=4* for TEA. (C and G) A549 cells were pre-treated with Qd (C), TEA (G) or a vehicle control as in (A). Cells were infected with rHAZV-eGFP and imaged 24 hpi using the IncuCyte Zoom system (scale bar = 200 µm). Representative images are shown. (D and H) Quantification of (C) and (G) as in Figure 2E, normalised to untreated cells (0 µM). *n=5* for TEA, *n=8* for Qd. ns, p>0.05; *, p<0.05; **, p<0.01, ****, p<0.001. Error bars represent standard deviation. To test for cell viability, cells were maintained in drug-containing medium for 24 hours at 37 °C and cell viability assessed by MTS assay. Cell viability (I and J) was normalised to cells treated with a vehicle control, Cell viability was normalised to cells treated with a vehicle control. ns, p>0.05, error bars represent ±S.D of 3x technical repeats.

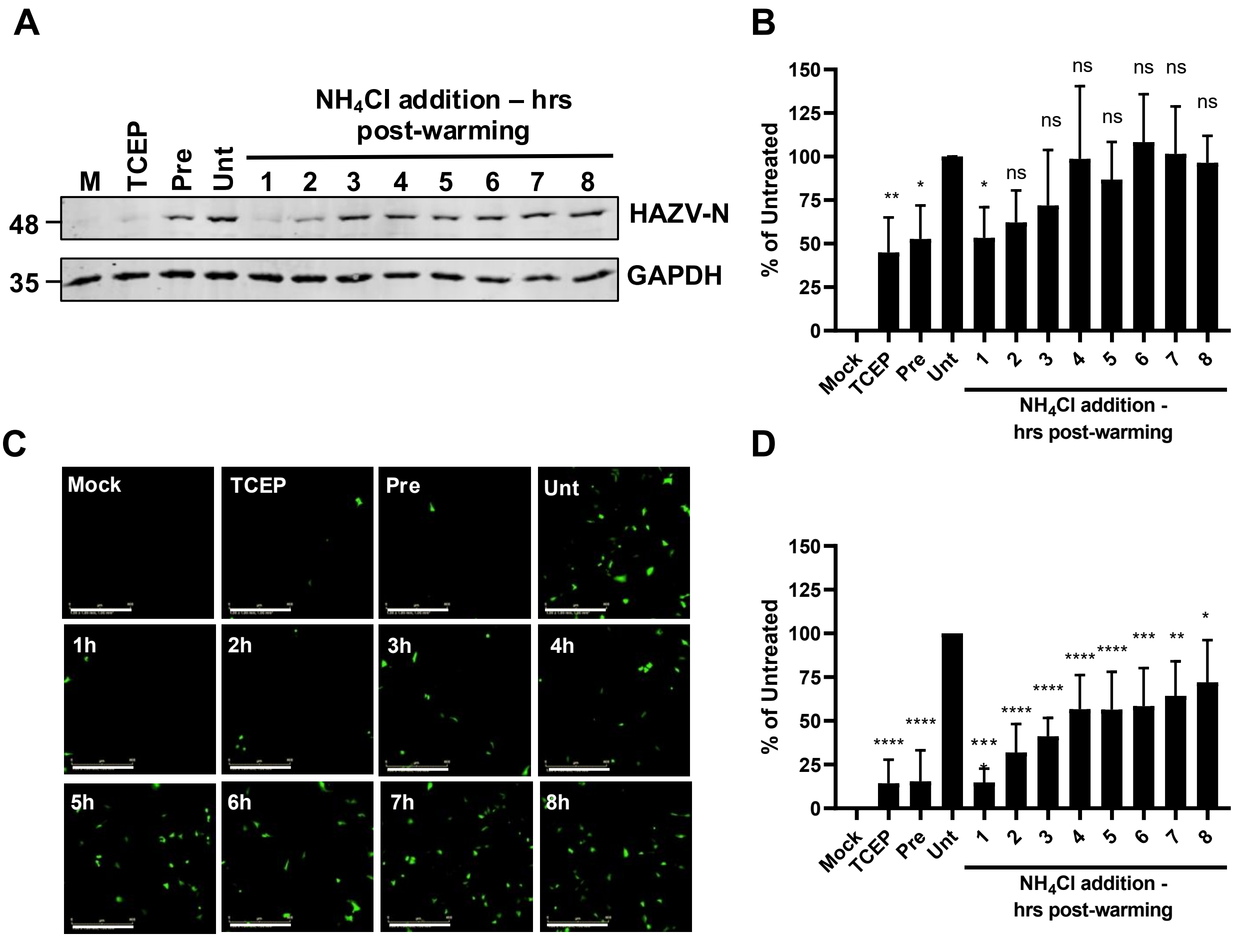

Supplementary fig. 5. HAZV is sensitive to neutralization of endosomal acidification at early stage of life cycle. (A) rHAZV-wt was bound to A549 cells in serum-free medium at 4°C for 1 hr (MOI 0.1). Inoculum was removed and cells were washed with PBS. Complete DMEM was pre-warmed to 37°C and added to cells to initiate internalisation (t=0). At the time points indicated, DMEM was removed and 20 mM NH_4Cl was added to cells. One well was treated with 10 mM TCEP prior to warming as a control for early internalisation. One was treated with NH_4Cl before and during infection as a control for drug activity (Pre) and one well was treated with an H_2O control (Unt). Cell lysates were collected at 24 hrs post-warming resolved by SDS-PAGE. HAZV-N expression was detected by western blot, with GAPDH used as a loading control. (B) Densitometry analysis of (A). Band intensity was normalised to untreated cells (Unt). *n=5*. (C) rHAZV-eGFP was bound to A549 cells at 4°C and time of addition assays were carried out as in (A). Images of infected cells were captured using the IncuCyte S3 (scale bar = 400 μm) at 24 hrs post-warming. (D) Quantification of (C) as in Figure 2E, expressed as a percentage of untreated cells (Unt). *n=7*. ns, p>0.05; *, p<0.05; **, p<0.01; ***, p<0.005; ****, p<0.001. Error bars represent standard deviation.

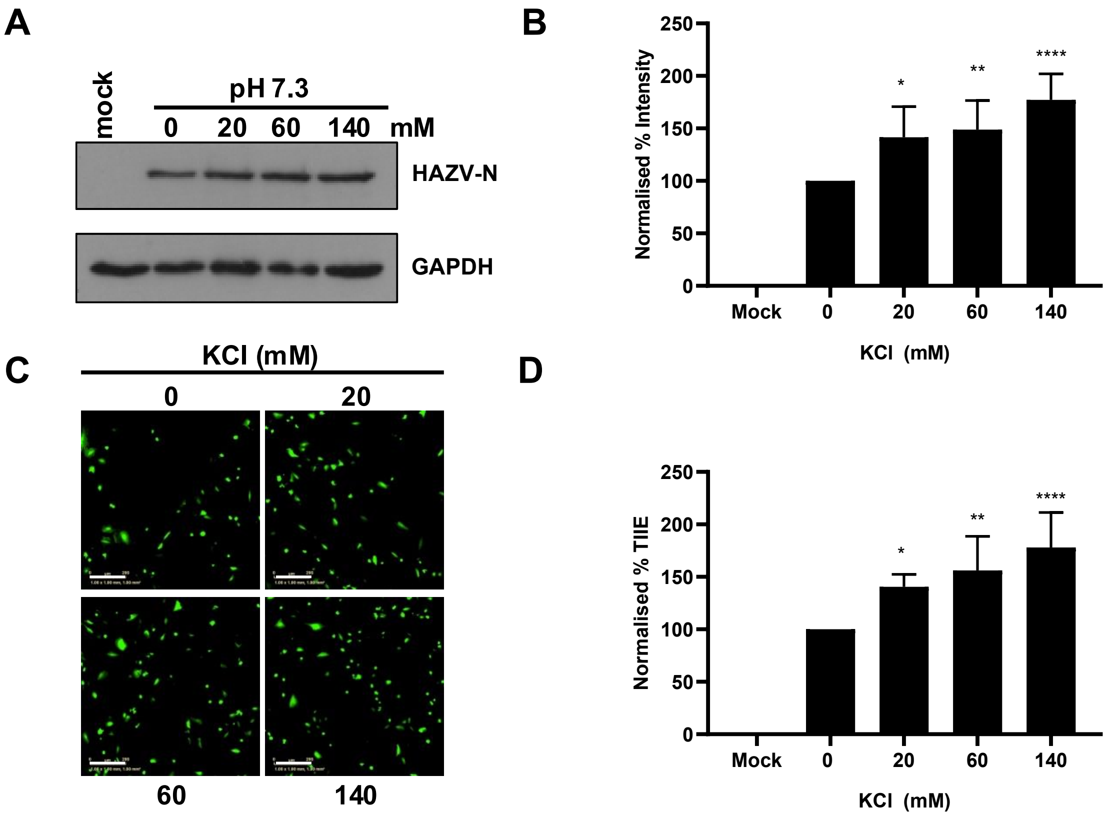

Supplementary fig. 6. HAZV infection is expedited at concentrations of K^+ encountered in early endosomes. (A) rHAZV-wt was incubated in pH 7.3 buffers plus 0, 20, 60 or 140 mM KCl for 2 hrs. Virus and buffer mix was diluted 1:11 in DMEM and added to A549 cells (MOI 0,1). Cell lysates were harvested 18 hpi and resolved by SDS-PAGE. HAZV-N expression was determined by western blot, with GAPDH used as a loading control. (B) Densitometry analysis of (A), normalised to pH 7.3 + 0 mM KCl. *n=6*. (C) rHAZV-eGFP was incubated in the indicated buffers as in (A) for 2 hrs. Cells were infected as in (A) and imaged 18 hpi using the IncuCyte S3 (scale bar = 400 µm.) Representative images are shown. (D) Quantification of (C) as in Figure 2E, normalised to pH 7.3 + 0 mM KCl. *n=5*. *, $p<0.05$; **, $p<0.01$. Error bars represent standard deviation.

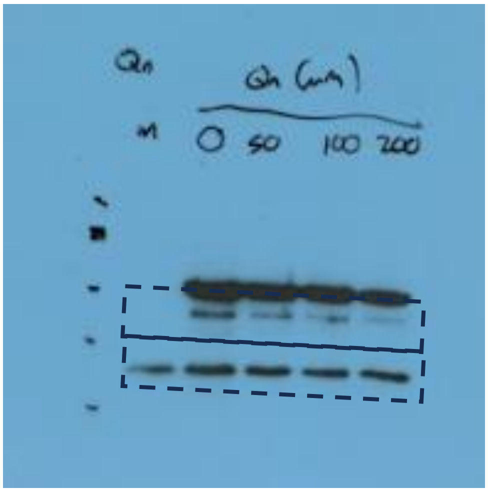

Supplementary fig. 7. Uncropped Western blots of those shown in Figure 2A. The dotted lines show the boundary of the cropped area.

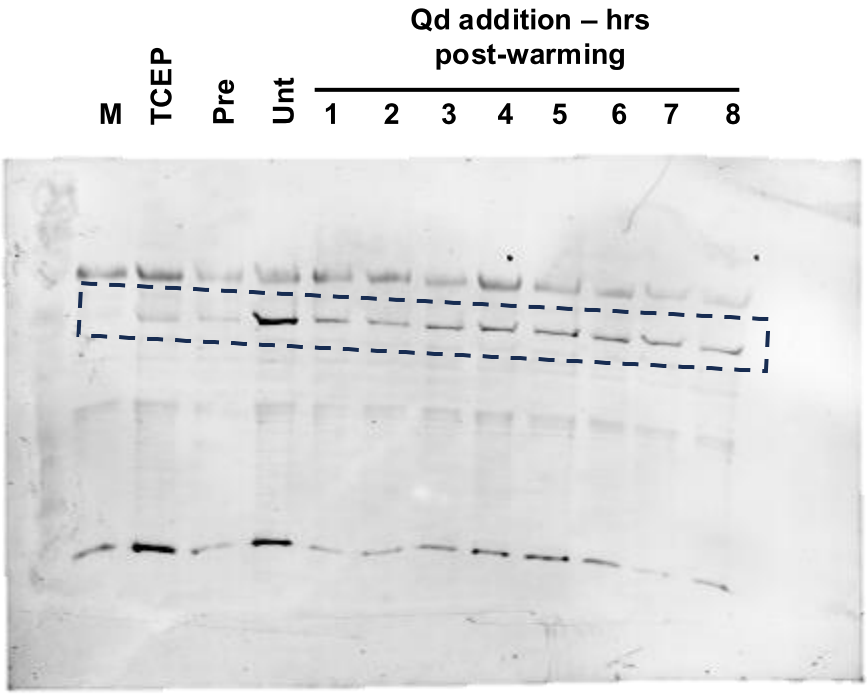

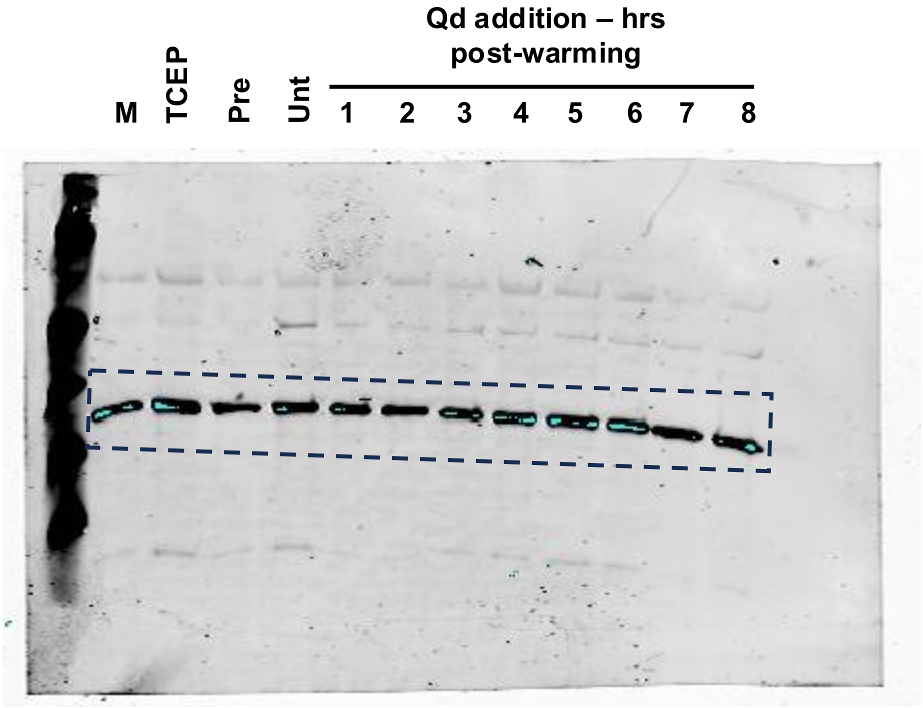

Supplementary fig. 8. Uncropped Western blots of those shown in Figure 3A. The dotted lines show the boundary of the cropped area.

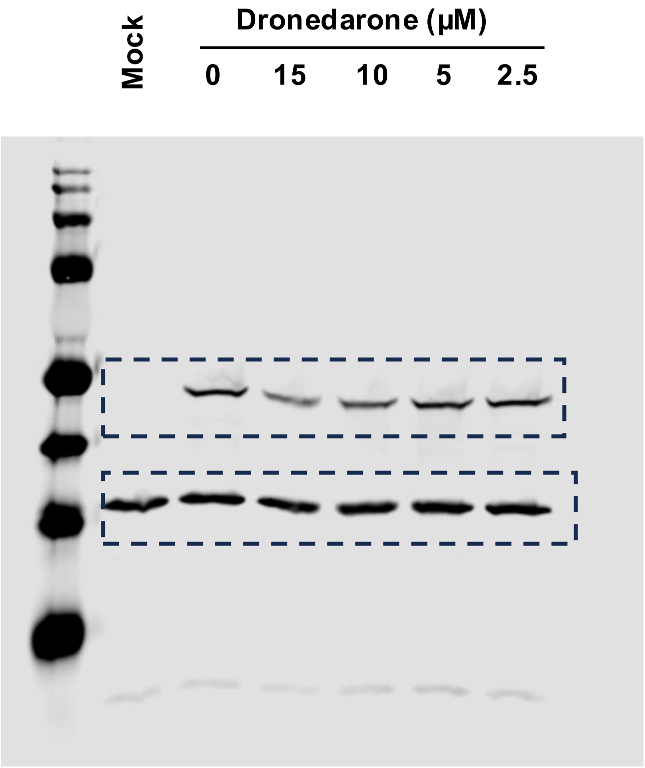

Supplementary fig. 9. Uncropped Western blots of those shown in Figure 4A. The dotted lines show the boundary of the cropped area.

A

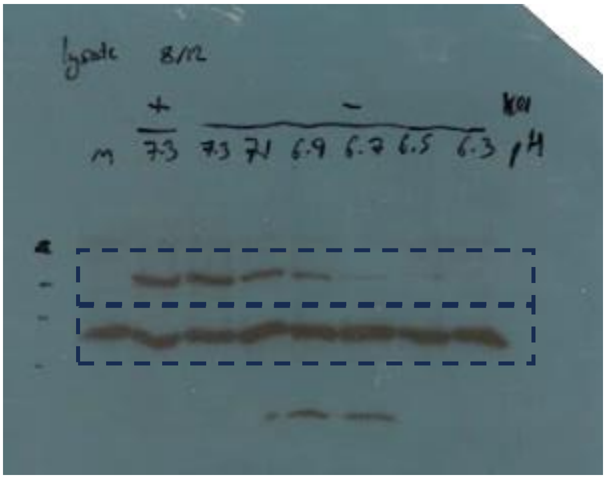

B

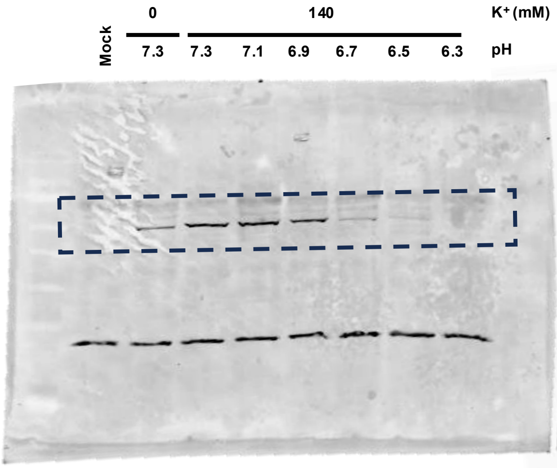

C

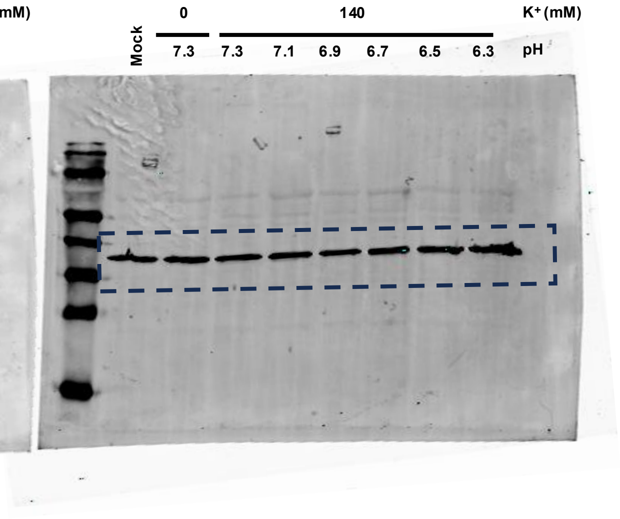

Supplementary fig. 10. Uncropped Western blots of those shown in (A) Figure 5A and (B-C) E. The dotted lines show the boundary of the cropped area.

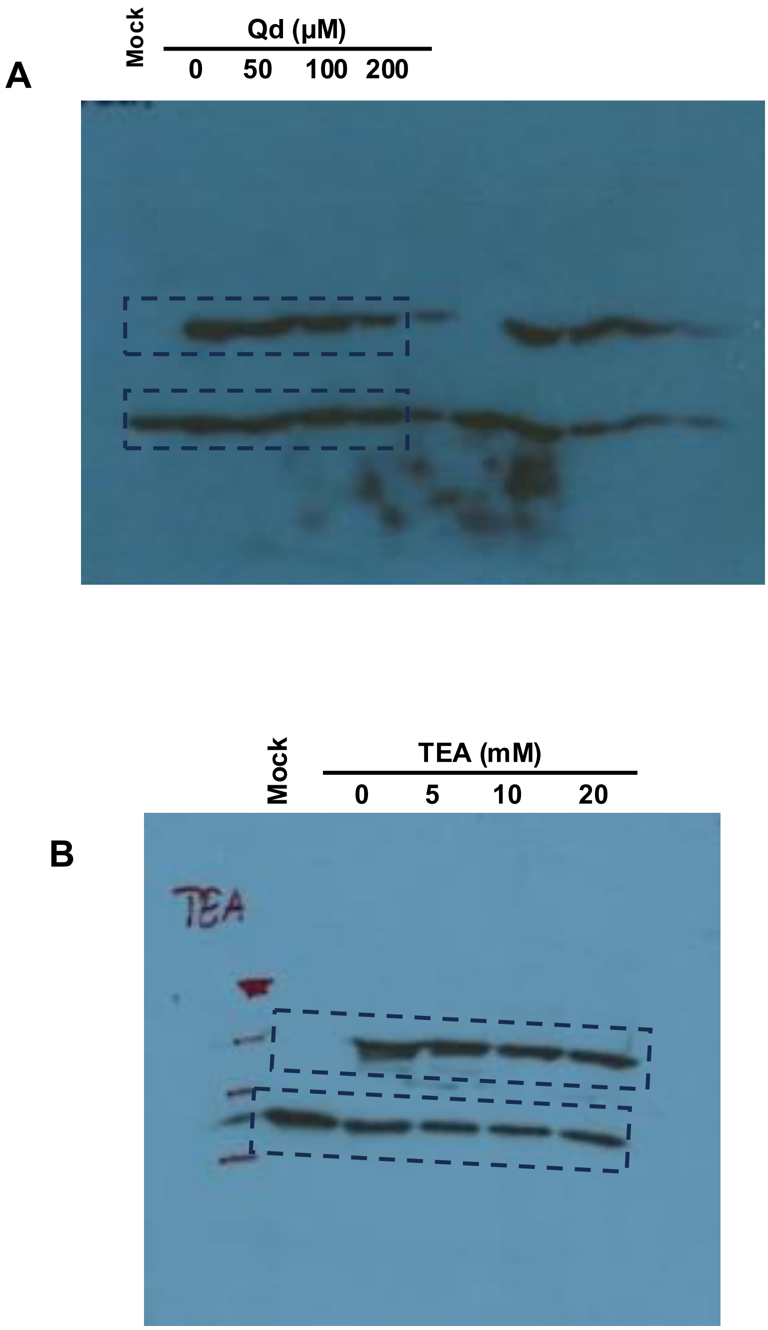

Figure S11. Uncropped Western blots of those shown in Figure S2A (A) and 2E (B). The dotted lines show the boundary of the cropped area.

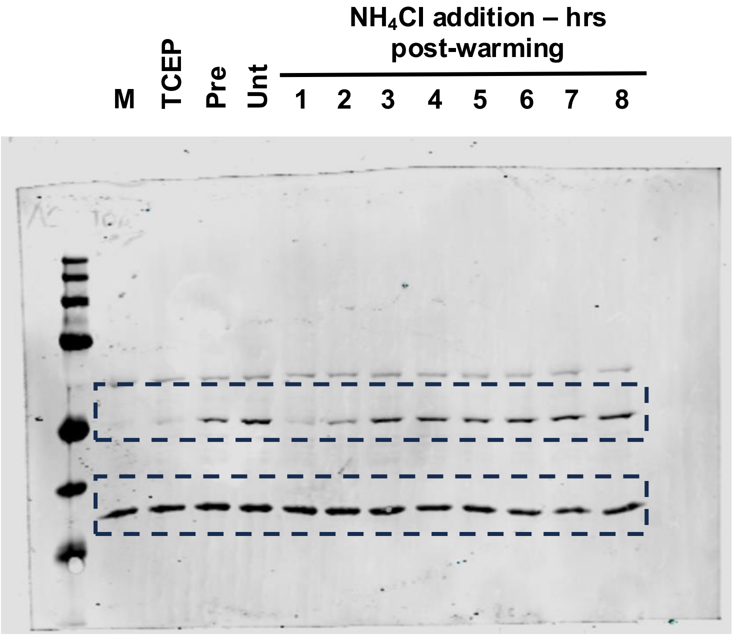

Figure S12. Uncropped Western blots of those shown in Figure S3A. The dotted lines show the boundary of the cropped area.

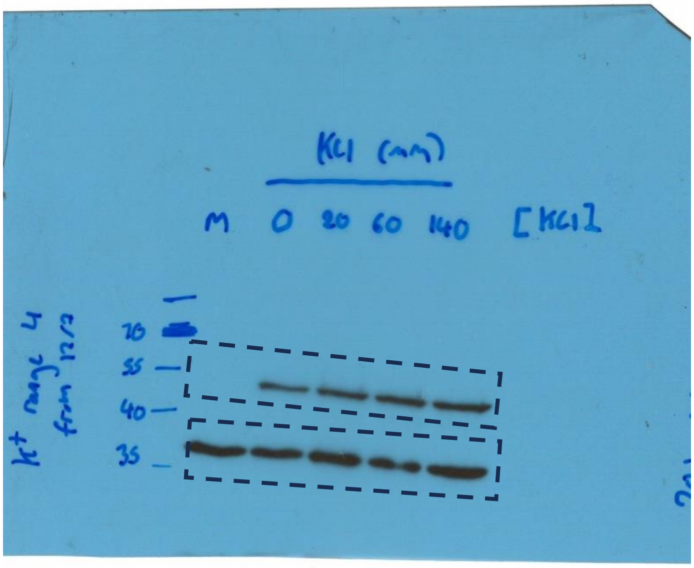

Figure S13. Uncropped Western blots of those shown in Figure S4A. The dotted lines show the boundary of the cropped area.

A
B

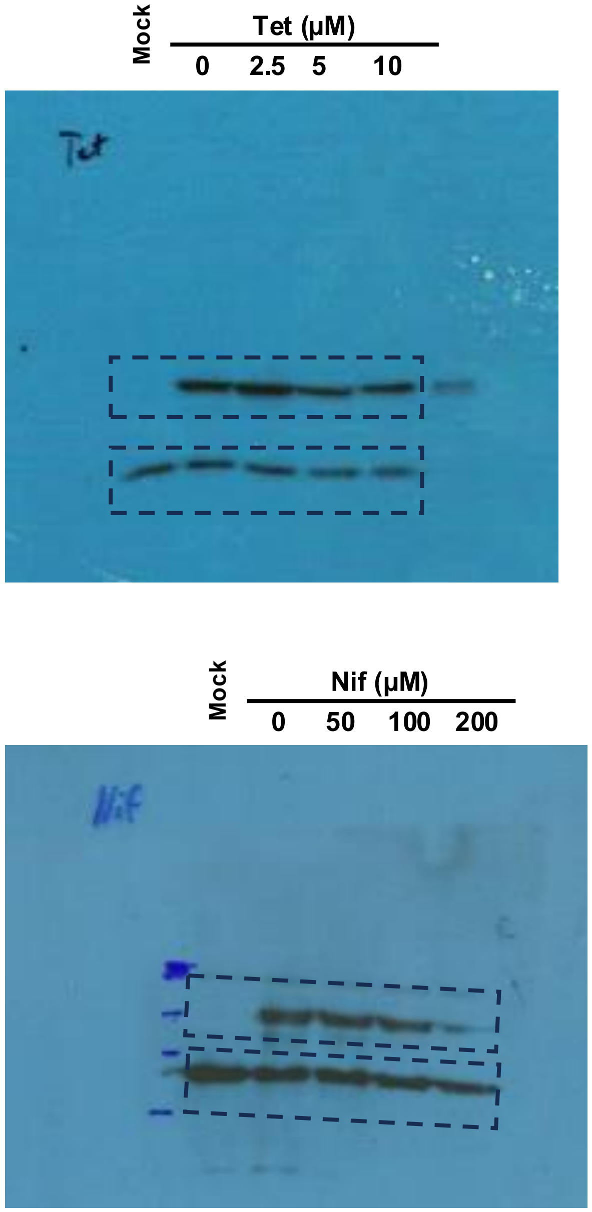

Figure S14. Uncropped Western blots of those shown in Figure S5A (A) and 5E (B). The dotted lines show the boundary of the cropped area.
